# Chemical-Assisted Microbially Mediated Chromium (Cr) (VI) Reduction Under the Influence of Various Electron Donors, Redox Mediators, and Other Additives: An Outlook on Enhanced Cr(VI) Removal

**DOI:** 10.3389/fmicb.2020.619766

**Published:** 2021-01-28

**Authors:** Zeeshanur Rahman, Lebin Thomas

**Affiliations:** ^1^Department of Botany, Zakir Husain Delhi College, University of Delhi, Delhi, India; ^2^Department of Botany, Hansraj College, University of Delhi, Delhi, India

**Keywords:** Cr(VI) reduction, chemical-assisted, electron donors, electron mediators, microbially mediated process

## Abstract

Chromium (Cr) (VI) is a well-known toxin to all types of biological organisms. Over the past few decades, many investigators have employed numerous bioprocesses to neutralize the toxic effects of Cr(VI). One of the main process for its treatment is bioreduction into Cr(III). Key to this process is the ability of microbial enzymes, which facilitate the transfer of electrons into the high valence state of the metal that acts as an electron acceptor. Many underlying previous efforts have stressed on the use of different external organic and inorganic substances as electron donors to promote Cr(VI) reduction process by different microorganisms. The use of various redox mediators enabled electron transport facility for extracellular Cr(VI) reduction and accelerated the reaction. Also, many chemicals have employed diverse roles to improve the Cr(VI) reduction process in different microorganisms. The application of aforementioned materials at the contaminated systems has offered a variety of influence on Cr(VI) bioremediation by altering microbial community structures and functions and redox environment. The collective insights suggest that the knowledge of appropriate implementation of suitable nutrients can strongly inspire the Cr(VI) reduction rate and efficiency. However, a comprehensive information on such substances and their roles and biochemical pathways in different microorganisms remains elusive. In this regard, our review sheds light on the contributions of various chemicals as electron donors, redox mediators, cofactors, etc., on microbial Cr(VI) reduction for enhanced treatment practices.

## Introduction

Chromium (Cr) is a pervasive toxin that inhabits almost every component of the environment including aerial, terrestrial, aquatic, and biological systems (Rahman and Singh, 2019). The nearly ubiquitous existence of this element is detrimental to natural establishments of Earth. Various environment and health protection agencies have considered Cr as a priority pollutant (DesMarias and Costa, 2019; Laxmi and Kaushik, 2020). The high magnitude of Cr contamination across the globe is estimated to pour risk on approximately 16 million people (Pure Earth, 2015). Both anthropogenic and natural events can contribute to the global Cr reservoir (Jeřábková et al., 2018; Coetzee et al., 2020; Tumolo et al., 2020). For example, industrial activities such as discharge of effluents and solid wastes from industrial energy production, manufacturing of refractories, stainless steel and chemical dye pigment production, chrome plating, treatment of wood, use of organic fertilizers and chemicals, waste and wastewater management, tanning, mining, etc., have created a widespread accumulation of Cr in their surroundings (Barnhart, 1997; Wilbur et al., 2012; Huang et al., 2019; Rahman and Singh, 2019). Cr is also used as catalyst, oxidizing agent and cooling agent with water (Saha et al., 2013). In another way, hastened dissolution of chromite and other minerals from natural reserves (i.e., serpentine soil and ultramafic rocks) as a natural event instigates the release of Cr into groundwaters upon suitable conditions (Gonzalez et al., 2005; Islam et al., 2020). Moreover, volcanic eruptions and forest fires also account for some Cr contamination in the environment (Viti et al., 2014).

The toxicity of Cr relies upon its oxidation states. Among commonly occurring states of Cr(0), Cr(III), and Cr(VI), the last species is viewed as more toxic than any other species (RoyChowdhury et al., 2018; Pechancová et al., 2019). Cr(VI) usually exists in the form of oxyanions of HCrO_4_^–^, CrO_4_^2–^, or Cr_2_O_7_^2–^ after reacting with oxygen (Rahman and Singh, 2019). The bioavailability and mobility of Cr(VI) are highly dependent on pH regulation, molecular oxygen (O_2_) availability, and the presence of organic matter and manganese oxides (MnO_2_) (Reijonen and Hartikainen, 2016; Shahid et al., 2017; Choppala et al., 2018). Cr speciation is also sensitive to soil redox potential (Eh), as the reduction and oxidation processes prevail at low and high Eh values, respectively (Xiao et al., 2015). Cr(VI) compounds are more toxic than Cr(III) compounds because of their high water solubility and mobility, whereas Cr(III) forms precipitation at body pH and does not exist in mobile species. Because of the structural similarity of chromate with sulfate, Cr(VI) can pass the cell membrane, but trivalent Cr fails to do so (Mukherjee et al., 2013). Further, the high oxidizing potential of Cr(VI) is also liable for its toxic effects in biological organisms (Sobol and Schiestl, 2012).

Cr(VI) displays genotoxic, carcinogenic, teratogenic, and mutagenic effects along with epigenetic profile silencing (Hu et al., 2016; DesMarias and Costa, 2019; Rager et al., 2019). As per the United States Environmental Protection Agency (US EPA), Cr(VI) is categorized as a “Group A” human carcinogen by the inhalation route of exposure (US EPA, 1998). Cr can induce phytotoxicity in plants by interfering with nutrient uptake and photosynthesis, generation of reactive oxygen species (ROS), lipid peroxidation, and altering the antioxidant activities (Shahid et al., 2017). Even elevated Cr(VI) concentrations can reduce the abundance of microbial communities by electron competing ability, inhibition of extracellular polymeric substances (EPSs) synthesis, and other effects including overproduction of ROS, protein and enzyme dysfunction, destruction of thiol and iron-sulfide cluster, inhibition of functional genes, nutrient assimilation and metabolic pathways, lipid peroxidation, DNA damage, etc. (Bhakta, 2017; Liu et al., 2017; Sun et al., 2019).

**FIGURE 1:**
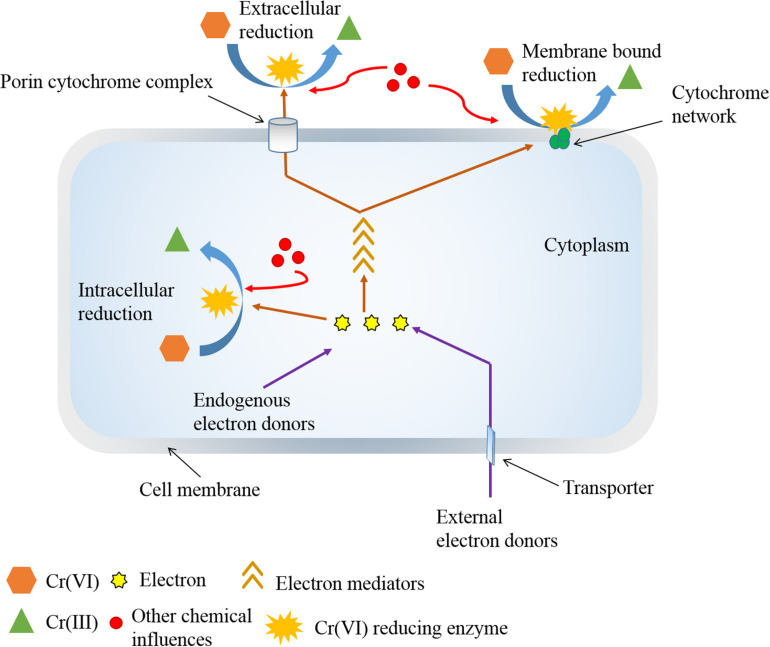
Role of various chemicals other than electron donors and mediators on enhanced Cr(VI) reduction.

Removal of Cr(VI) from the environment is challenging. There are many methods such as ion exchange, electrochemical precipitation, solvent extraction, membrane separation, evaporation, foam separation, ultrafiltration, electrodialysis, cementation, biosorption, and reduction (Mukherjee et al., 2013). However, the conventional reduction and precipitation methods of Cr(VI) removal utilize large amounts of chemicals and generate enormous toxic sludge (Mukherjee et al., 2013). The recent possible removal measures rely on the implementation of biotechnological approaches, considering ecofriendly and cost-effective approaches (Fernández et al., 2018; Jobby et al., 2018). Especially, Cr(VI) reduction by microorganisms is being recognized as an eventual treatment. Despite successful outcomes, certain disadvantages such as low efficiency and poor compatibility due to limited availability of electron donors and other inducers often hamper the prominence of Cr(VI) bioremediation in large-scale implementation (Malaviya and Singh, 2016; Beretta et al., 2019; Wang et al., 2020). These limitations can be subdued by introducing low concentrations of suitable chemicals in the medium (Figure 1). But such chemical integration demands a detailed understanding of additive-influenced mechanisms in credible microorganisms. Considering this significance, this review recognizes and emphasizes the experimental studies of influences of electron donors, electron mediators, and other chemical additives on microbial Cr(VI) reduction for the enhanced Cr(VI) removal approaches.

## Cr(VI) Reduction by Microorganisms

Several different microorganisms have adopted various strategies to counter-effect the toxicity of Cr(VI). Among different methods, enzymatic reduction of Cr(VI) into Cr(III) by microorganisms is the best characterized mechanism for its bioremediation (Singh et al., 2008). All Cr(VI)-resistant microbes cannot reduce Cr(VI). However, Cr(VI) resistance is a common phenomenon in all Cr(VI) bioreducers, which delivers proficiency in detoxification process (Singh et al., 2008; Thatoi et al., 2014). The catalysis of Cr(VI) reduction can be demonstrated using chromosome or plasmid-encoded non-specific enzymes (Cervantes and Campos-García, 2007; Pradhan et al., 2016; Baldiris et al., 2018). These enzymes are mainly oxidoreductases such as chromate reductases (ChrA and YieF), NADH-dependent nitroreductase, iron reductase, quinone reductases, hydrogenases, NADH/NADPH-dependent flavin reductases, and NADPH-dependent reductases (Puzon et al., 2002; Kwak et al., 2003; Ackerley et al., 2004). Even many microorganisms exhibited Cr(VI) reduction using reductases with multiple substrate specificity (Table 1). Cr(VI) reduction enzymes have shown inductive or constitutive expression in different microorganisms (Ibrahim et al., 2012; Chai et al., 2018).

**TABLE 1:** Cr(VI) reduction in different microorganisms using reductases with multiple substrate specificity.

Microorganisms	Enzymes	Other substrates	References
*Alishewanella* sp. WH16-1	Selenite reductase (CsrF)	Selenium (Se)	Xia et al. (2018)
*Leucobacter* sp.	Dihydrolipoyl dehydrogenase	Dihydrolipoamide	Sarangi and Krishnan (2016)
*Caldicellulosiruptor saccharolyticus*	Ni-Fe hydrogenase	Hydrogen	Bai et al. (2018)
*Vibrio harveyi*	Nitroreductase (NfsA)	Nitroaromatic compounds	Kwak et al. (2003)
*Staphylococcus*	NfoR	Flavin and FMN	O’Neill et al. (2020)
*aureus* LZ-01			
*Escherichia coli*	NemA	Glycerol trinitrate and pentaerythritol tetranitrate	Robins et al. (2013)
*Streptomyces violaceoruber* strain LZ-26-1	Thioredoxin operon	Thioredoxin	Chen et al. (2014)

Biological Cr(VI) reduction activities can occur at extracellular, cell membrane, and intracellular locations in aerobic and anaerobic conditions (Cervantes et al., 2001; Cervantes and Campos-García, 2007). Extracellular Cr(VI) reduction is regulated by soluble (cytoplasmic) proteins exported to the extracellular medium by an energy-intensive process (Cheung and Gu, 2007; Elangovan et al., 2010; Das et al., 2014). This mechanism is accustomed to protect microbes from the damaging effects of Cr(VI) by minimizing its active intracellular transport (Wani et al., 2018). In anaerobic reduction, microorganisms may use Cr(VI) as the terminal electron acceptor in electron transport system associated with membrane enclosed regions (Ramírez-Díaz et al., 2008). Consecutively, the transformation of Cr(VI) into Cr(III) can also occur spontaneously in microorganisms by chemical reactions using different intracellular or extracellular compounds, metabolic end products, and intracellular reductants of ascorbate glutathione, cysteine, and hydrogen peroxide (H_2_O_2_) (Donati et al., 2003; Viti and Giovannetti, 2007; Poljsak et al., 2010; Thatheyus and Ramya, 2016).

The enzymatic reduction of Cr(VI) to Cr(III) can also yield variable concentrations of ROS, which may or may not involve in the formation of reactive intermediates (Cheng et al., 2009; McNeill et al., 2012; Baldiris et al., 2018). However, different reductase enzymatic activities possess some capacity to alleviate the effects of ROS. Accordingly, homologous enzymes involved in catalyzing the electron movement from electron donors to reduce Cr(VI) are classified into class I (tight) and class II (semitight) reductases (Thatoi et al., 2014; Baldiris et al., 2018). The class I reductases catalyze one-step electron reduction of Cr(VI) to form the highly unstable Cr(V) intermediate. The tendency of this reactive intermediate to oxidize into Cr(VI) by donating electrons to molecular oxygen generates ample ROS. However, class II chromate reductases are two electron reducers of Cr(VI), in which the formation of Cr(III) proceeds without forming a Cr(V) intermediate. This results in much lesser generation of ROS during the reduction process (Thatoi et al., 2014). Moreover, quinone reductase activity can further neutralize the effects of ROS to some extents (Cheung and Gu, 2007; Thatoi et al., 2014). Apart from this, chromate-resistant microorganisms also tend to stimulate the efflux system, SOS response of DNA damage repair, ROS scavenging enzymes (catalase, superoxide dismutase, etc.), and non-enzymatic antioxidants (vitamin C and E, carotenoids, thiol antioxidants, and flavonoids) for inactivating ROS-mediated oxidative stress (Ackerley et al., 2006; Cervantes and Campos-García, 2007; Flora, 2009).

## Electron Donors

In general, electron donors release electrons during cellular respiration accompanied by the release of energy. But such cellular reaction in microorganisms also enables biological treatment by providing electrons to Cr(VI) (Ceci et al., 2019; Truskewycz et al., 2019). Even suitable electron donors can facilitate the enhancement of reduction activity (Poopal and Laxman, 2009; Alam and Ahmad, 2012; Ibrahim et al., 2012; Shi et al., 2012). The requirement of electron donor is indispensable for both microbial growth and Cr(VI) reduction (Poopal and Laxman, 2009). Most microbial species capable of Cr(VI) reduction are heterotrophic and need such additional nutrition from external sources during the reduction process (Ancona et al., 2020). Certainly, some endogenous reserves can also serve electrons to Cr(VI) during its bioreduction (McLean and Beveridge, 2001; Ray et al., 2018).

**FIGURE 2:**
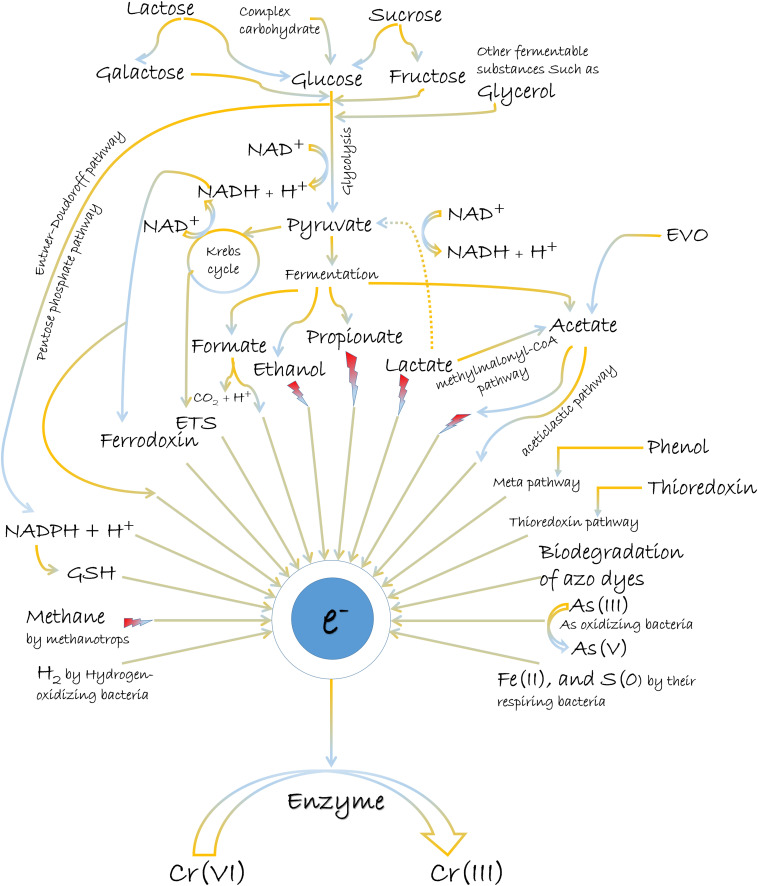
The general mechanisms of chemical-assisted microbially mediated Cr(VI) reduction.

The most common electron donors for Cr(VI) reduction are organic molecules, although reports on inorganic matters as reducing equivalents also exist (Figure 2). Usually, the abundant oxygen-containing functional groups (C–O, CO–OH, C–OH, and C–O–R) in organic matters present the utility as electron donors (π electrons) (Choppala et al., 2016; Shaheen et al., 2019; Xu et al., 2019). Glucose, fructose, lactose, pyruvate, lactate, citrate, glycerol, acetate, formate, NADH/NADPH, reduced glutathione, etc., are the various popular electron donors for Cr(VI) reduction (Garbisu et al., 1998; Poopal and Laxman, 2009; Murugavelh and Mohanty, 2013; Salamanca et al., 2013; Ahemad, 2014; Mala et al., 2015). Many such examples for the treatment of Cr(VI) can be further studied from Table 2. Among them, the widespread electron donor implicated in Cr(VI) reduction process is NAD(P)H, which is usually associated with intracellular reduction (Robins et al., 2013; Tan et al., 2020). Even many chromate reductase enzymes exhibited NAD(P)H-dependent properties, where the attachment of dehydrogenase molecule to enzyme is crucial for a feasible reaction. The reductase enzyme in *Alishewanella* sp. possessed Arg^13^ and Gly^113^ residues for the cobinding of NAD(P)H and Cr(VI) (Xia et al., 2018). Another Flavin mononucleotide (FMN)-containing chromate reductase of *Gluconacetobacter hansenii* (Gh-ChrR) induced structural rearrangement of active site during the binding of chromate anion and NADH. With this rearrangement, both species could bind simultaneously for efficient enzyme cycling, of which binding site otherwise overlapped with the electron donor (Jin et al., 2012). Also, microbial Cr(VI) reduction using glucose as the donor source is very prominent (Camargo et al., 2003;

**TABLE 2:** Enhancement of Cr(VI) reduction using different electron donors by different microorganisms.

Microorganisms	Electron donors	Aerobic/anaerobic	Concentrations of electron donor (a) and Cr(VI) (b)	Chromium reduction (%)		References
				Without electron donor	With electron donor	
*Halomonas chromatireducens* AGD 8–3	Acetate	Aerobic	(a) 20 mM	0	>90	Shapovalova et al., 2009
			(b) 30 mg/L			
*Halomonas smyrnensis* KS802	Galactose	Aerobic	(a) 4%	82.5	100	Biswas et al., 2018
			(b) 2 mM			
*Bacillus methylotrophicus*	Reduced glutathione	Aerobic	(a) 20 mM	89.8	94.5	Mala et al., 2015
			(b) 200 μM			
*Ochrobactrum intermedium* Rb-2	Gluconate	Aerobic	(a) 1%	<95	99	Batool et al., 2012
			(b) 1,000 mg/L			
*Enterobacter* sp. DU17	Glucose	Aerobic	0.2%	59	100	Rahman and Singh, 2014
	fructose		100 mg/L		100	
*Pseudomonas aeruginosa* CRM100	Citrate	Anaerobic	(a) 4.0 g/L	0	99.73	Salamanca et al., 2013
	glycerol		(b) 100 mg		88.4	
*Pelosinus* sp. HCF1	Lactate	Anaerobic	(a) 20 mM	<12	>90	Beller et al., 2013
			(b) 45 μM			
*Arthrobacter* sp. LLW01	Lactate	Aerobic	15 mM	0	80	Field et al., 2018
			50 μM			
Microbial consortium	H_2_: CH_4_	Anaerobic	1:1 ratio	0	95.5	He et al., 2020
			10 mg/L Cr(VI)			
*Flexivirga alba* ST13T	Molasses	Aerobic	0.4%	<10	>95	Ikegami et al., 2020
			0.5–0.6 mg/L			
*Stenotrophomonas* sp. WY601	Lactose, fructose, and glucose	Anaerobic	2%	0	68–80	Liu et al., 2019a
			2,500 mg/L			
*Bacillus* sp. M6	Glycerol	Aerobic	0.1 mM/L	3.4	69.2	Li et al., 2019a
			20 mg/L			
Microcosms	Yeast extract	Aerobic	200 mg/L	<10	100	Ancona et al., 2020
			1,000 μg/L			
*Bacillus* sp. CRB-B1	Fructose	Aerobic	10 g/L	0	89.54	Tan et al., 2020
			100 mg/L			
*Bacillus cereus*	NADH,	Aerobic	1 g/L	40	91.52,	Murugavelh and Mohanty, 2013
	NADPH		60 mg/L		96.54	
*Lysinibacillus fusiformis* ZC1	Acetate, NADH	Aerobic	1%	21	92.4 92.0	He et al., 2011
			0.1 mM			
*Intrasporangium* sp. Q5–1	Acetate	Aerobic	0.5 mM/L	75	95	Yang et al., 2009
			0.2 mM			
*Oceanobacillus oncorhynchi* W4	Glycerol	Aerobic	3 g/L	<10	72.3	Zheng et al., 2019
			10 mg/L			

Megharaj et al., 2003; Satarupa and Paul, 2013; Ziagova et al., 2014). Glucose, being the most easily metabolized carbon source, can deliver maximum electrons for Cr(VI) reduction. The synthesis of volatile fatty acids (VFAs) during glucose fermentation provides additional sources of electron donors, which can also be attributed for the improved performance of Cr(VI) reduction (Zheng et al., 2019). Furthermore, glucose exhibits indirect action on Cr(VI) reduction by promoting microbial growth (Leita et al., 2011). However, the role of glucose as the most suitable electron donor is not consistent for all Cr(VI) reducer. Anaerobic process by *Pannonibacter phragmitetus* LSSE-09 promoted Cr(VI) reduction more in presence of acetate, lactate, and pyruvate rather than glucose (Xu et al., 2011). Another anaerobic strain, *P. phragmitetus* BB also exhibited more reduction of Cr(VI) in presence of lactate than many other electron donors. Metabolic analyses of strain BB elucidated that the presence of Cr(VI) upregulated pyruvate dehydrogenase and lactate dehydrogenase, and these dehydrogenases preferentially transferred the cellular electrons to extracellular Cr(VI) over other electron acceptors (Chai et al., 2019). In another study, anaerobic suspensions of *Pelosinus* sp. HCF1 reduced chromate in the presence of lactate as a sole electron donor (Beller et al., 2013). Lactate fermentation to acetate and propionate through methylmalonyl-CoA pathway also broadened the availability of electron donors in the system (Beller et al., 2013). Bill et al. (2019) also corroborated the evidence of lactate fermentation into simpler metabolites and further degradation into CO_2_. In this process, Cr(VI) removal was basically associated with the breakdown of carboxylic acids. However, lactate as an electron donor also causes a significant abiotic Cr(VI) reduction due to the formation of a lactate–Cr(VI) complex or sodium lactate syrup that functions as a non-specific reductant (Brodie et al., 2011). Fungal metabolites such as salicylate, tartrate, and citrate produced by a metalliferous *Aspergillus tubingensis* Ed8 were also stimulators for Cr(VI) reduction (Coreño-Alonso et al., 2009). However, non-fermentable substrates such as citrate, butyrate, and acetate as electron donors have shown usually lower effects for enhanced Cr(VI) removal rates (Orozco et al., 2010). Nevertheless, the contribution of acetate as the most suitable electron donor for Cr(VI) reduction in certain microorganisms is also not deniable. The accumulation of acetate as one of the fermentation product has greatly influenced Cr(VI) reduction in the anaerobic system (Bai et al., 2018; Zheng et al., 2019; Zhang et al., 2020). In an investigation, addition of Cr(VI) shifted the amounts of fermentation products toward more oxidative form, i.e., acetate (∼2.5 times) (Sharma, 2002). The plausible reason of this shift toward acetate formation after Cr(VI) exposure is likely to secure more NADH molecule that can be channeled toward bioreduction process. Mass balance reaction also supported the reason, which derived more loss of NADH molecules by butyrate and lactate formation than acetate synthesis (Sharma, 2002). Occasionally, acetoclastic pathway by Archaea instigated the release of electrons from acetate for Cr(VI) reduction (Hu et al., 2018). Aerobic bacterial strains, *Lysinibacillus fusiformis* ZC1 and *Intrasporangium* sp. strain Q5-1 also consumed acetate as the most suitable electron donor for Cr(VI) reduction in reports of He et al. (2011) and Yang et al. (2009), respectively.

The bacterium *Pseudomonas aeruginosa* CCTCC AB91095 offered simultaneous removal of phenol and Cr(VI), where the degradation of the former compound supported Cr(VI) reduction (Song et al., 2009). Supposedly, meta-pathways maintained the requirement of electrons from phenol biodegradation for Cr(VI) removal (Chen et al., 2003; Song et al., 2009). The bacterium, *Brevibacterium casei* employed azo dye acid orange 7 (AO7) as an electron donor for Cr(VI) reduction by coupling with dye decolorization under nutrient-limiting conditions (Ng et al., 2010). Another reactive black-5 azo dye was also proposed to serve electron for Cr(VI) reduction by bacterial strains (Mahmood et al., 2013). Chen et al. (2014) identified a new pathway for Cr(VI) reduction by *Streptomyces violaceoruber* LZ-26-1, which involved thioredoxin as an electron donor following its reduction using thioredoxin reductase. However, the main supply of electrons to thioredoxin was originated from NADPH molecules.

The utilization of electron donors for Cr(VI) reduction is species-dependent, and the reduction rate in specific microorganisms also varies for different sources (Das et al., 2014; Zhang et al., 2018). The electron donor that is effective in catalyzing Cr(VI) reduction for one microorganism might not be as useful for other species. It is also highly probable that multiple reduction pathways of variable efficiencies can be operated for different electron donors in different species. The capacity of electron donation of different donor compounds also relies on a mobile cytochrome electron carrier, quinone pool, or a dehydrogenase activity, where the measures of decreased potential differences between donor and acceptor compound states matter (Kracke et al., 2015). However, if the available electron donors are not suitable for any microorganism, adverse impacts on both growth and reduction process are noticeable. For example, Cr(VI) can be more toxic to microbes, when ethanol or butyrate is the sole electron donor as compared to more favorable glucose or lactate (Field et al., 2018). The cells in aerobic systems cannot gain enough energy from butyrate or ethanol for growth. Therefore, the availability of electrons for Cr(VI) reduction remains insufficient. Moreover, the presence of ethanol decreases the cell viability by shearing biological membrane, and metabolically active cells become more susceptible to alcohol and Cr(VI) uptake and their toxicity. Nevertheless, some anaerobic conditions have utilized the route of oxidation of ethanol by sulfate reducing bacteria for Cr(VI) removal (Pagnanelli et al., 2012; Cirik et al., 2013).

In recent years, microbial synergy has been used to couple various electron donors for effective Cr(VI) reduction process (Lu et al., 2020; Shi et al., 2020a). The outcomes have demonstrated obvious improvement in Cr(VI) reduction. The utilization of both hydrogen and methane (CH_4_) as dual electron donors involving microbial consortia of autohydrogenotrophic bacteria (e.g., *Hydrogenophaga*, *Thiobacillus*, and *Acetoanaerobium*), CH_4_–metabolizing microorganisms (e.g., *Methanobacterium* and *Methanosaeta*), and heterotrophic Cr(VI) reducers (e.g., *Geobacter*, *Spirochaetaceae*, *Delftia*, and *Anaerolineaceae*) promoted Cr(VI) reduction in different bioreactors (He et al., 2020, 2021). However, microbial anaerobic Cr(VI) reduction using hydrogen (H_2_) or CH_4_ as an individual electron donor also persists, but the capacity of removal is limited in different aspects (Chung et al., 2006, 2007; Lai et al., 2016; Luo et al., 2019). In a CH_4_-based membrane biofilm reactor (MBfR), metagenomics analysis showed that oxidation of CH_4_ by a sole anaerobic methanotroph, *Candidatus* “Methanoperedens,” in the biofilm was responsible for Cr(VI) reduction (Luo et al., 2019). Another MBfR exhibited relative abundance of three genera *Methylocystis*, *Meiothermus*, and *Ferruginibacter*, which was coupled with CH_4_ oxidation, and Cr(VI), Se(VI), and SO_4_^2–^ reduction (Lv et al., 2018). In other ways, S(0) or Fe(0) can also be used as inorganic electron donors for biologically Cr(VI) reduction employing microbial synergism. Shi et al. (2019) found that VFAs produced by Fe(0) or S(0) oxidizing bacteria (e.g., *Thiobacillus* or *Ferrovibrio*) could be utilized by Cr(VI) reducer (e.g., *Geobacter* or *Desulfovibrio*). Another sulfur-based mixotrophic Cr(VI) reduction process involved autotrophic sulfur oxidation and heterotrophic chromate reducing bacteria such as *Desulfovibrio* and *Desulfuromonas* (Zhang et al., 2020). The coupling of different processes such as microbial sulfur cycle, Fe(III)/Fe(II) transformation, phenol degradation, and Cr(VI) reduction by microbial aggregates involving bacteria such as *Desulfovibrio*, *Comamonas*, *Ochrobactrum*, and *Thiobacillus* offered effective and simultaneous removal of multiple contaminants including Cr(VI) and also facilitated the reduction of the reoxidized Cr(III) (Zhao et al., 2020b). Recently, the mixed biogas forms, CH_4_ and hydrogen, regulated by bacteria such as *Geobacter* and *Methanobacterium* in a microbial fuel cell (MFC) promoted Cr(VI) reduction by Cr(VI) reducers (e.g., *Hydrogenophaga*, *Thiobacillus*, *Geobacter*, and *Anaerolineaceae*) coupled with hydrogen-oxidizing bacteria (e.g., *Hydrogenophaga* and *Thiobacillus*) and CH_4_-oxidizing bacteria (e.g., *Methanobacterium* and *Methanosaeta*) (He et al., 2021).

Microbes are further known to utilize composite matters or combinations of donor compounds for Cr(VI) reduction (Figure 2; Shi et al., 2012; Mala et al., 2015; Huang et al., 2019). Abundant natural substances such as cellulose, hemicellulose, pectin, starch, and xylose can be the bulk sources of reducing equivalents like NADH and reduced ferredoxin during the metabolic route of glucose fermentation (Bai et al., 2018). Cellulosic waste that can be biodegraded to other carbon sources such as sugars, organic acids, and alcohols can readily stimulate bacterial growth and the subsequent reduction of Cr(VI) (Thomas et al., 2016; Field et al., 2018). This was also supported by an observation where Cr stress situation enhanced production of cellulase enzyme for the hydrolysis of cellulose (Aslam et al., 2019). Another study measured the coupling of cellulose and cellulose-degrading bacterium, *Cellulomonas* strain Lsc-8 in an MFC to reduce Cr(VI), and generated electricity simultaneously (Cao et al., 2020). Emulsified vegetable oil (EVO) and molasses as carbon sources have also influenced the biological metabolism to promote Cr(VI) reduction (Michailides et al., 2015; Wen et al., 2017). EVO being an oil-in-water emulsion operated as a slowly released electron donor by fermenting into acetate and hydrogen (Wen et al., 2017). EVO has advantages over other soluble substrates for Cr(VI) reduction, as it can provide long-term electrons for the biological process by inhibiting rapid biodegradation, and downgradient migration (Wen et al., 2017). Likewise, another solid substrate, cellulose-rich materials as a biodegradable meal box (BMB) also exhibited property of slowly releasing electrons for Cr(VI) reduction (Li et al., 2016). Molasses as an economical and readily available carbon source have offered more efficient Cr(VI) reduction than by many other sugars. The superiority of molasses over other carbon sources in Cr(VI) reduction can be attributed to its diverse constituents such as nitrogenous substances, vitamins, trace elements, and a large amount of different sugars (Smith et al., 2002; Field et al., 2013). Also, the presence of more easily utilizable sugars in molasses can be presumably more effective than pure sucrose for electron donating capability (Field et al., 2013). The phenolic hydroxyl and carbonyl groups of reducing substances such as flavonoids in molasses might also supply the electrons to the Cr(VI) (Okello et al., 2012; Chen et al., 2015). Complex nutrient media such as yeast extract and peptone can act as energy substances to support natural microbial communities, which rapidly increase the biological Cr(VI) reduction (Ancona et al., 2020; Dey and Paul, 2020). Possibly, such constituents in different culture media have always influenced the Cr(VI) reduction process by electron donating property as well. In contrary, some organic constituents in complex matters can interfere with the bioavailability of Cr(VI) for its reduction (Dey and Paul, 2020). Also, the competition of electron donors for the reduction of other co-contaminants such as SO_4_^2–^, NO_3_^–^, and Se(VI) might often impede with enhanced Cr(VI) reduction (Zhong et al., 2017; Lv et al., 2018). But the advantage lies as the oxidation of organic compounds preferentially donates electrons to Cr(VI) over many other acceptor molecules considering its high reducing potential (Cirik et al., 2013).

## Electron Mediators (Or Electron Shuttles)

Extracellular reduction may require some passage capacity for intracellular reducing equivalents to approach exterior Cr(VI). Such pathway raises the value of electron mediators (or electron shuttle) for reduction outside the cell (Bai et al., 2018). Mediators can reversibly reduce and oxidize to shuttle electrons from cellular boundary to terminal electron acceptor [i.e., Cr(VI)] (Han et al., 2016; Tanaka et al., 2018; Cheng et al., 2019). In this process, complexes such as porin-cytochrome located on the bacterial membrane can provide an interface for the transfer of intracellular electrons using redox mediators (Richardson et al., 2013). Mediators have a role in accelerating the reducing activity rather than promotion effect on the Cr(VI) reduction (Huang et al., 2019). Because such additives can prominently speed up the electron transfer rate (Cheng et al., 2019). Such chemical reaction is feasible when the standard redox potential of an electron mediator lies between the standard redox potentials of two half-reactions (Kavita and Keharia, 2012). In this conversion, electrons can readily shuttle from low-potential electron donors to mediators and then from low-potential mediators to the terminal electron acceptor, i.e., Cr(VI). Consequently, mere contacts among such components are sufficient for the catalysis of the Cr(VI) reduction (Voordeckers et al., 2010). The most suitable range of redox potential for mediators to succeed Cr(VI) reduction lies between −0.320 and 1.28 V, where the previous value belongs to cofactor NADPH, and the latter for chromate (Kavita and Keharia, 2012). Some redox mediators are also reported to overcome thermodynamic barriers of redox conversions and steric hindrance of the reactants (Sun et al., 2015; Dai et al., 2016). Appropriate concentrations of electron donors are also a crucial factor for the regeneration of mediators, whereas the absence of suitable electron donors may substantially hamper the activity of mediators (Chen et al., 2011; Xafenias et al., 2013; Bai et al., 2018). Many investigations identified that electron mediators could exhibit a concentration-dependent effect on Cr(VI) reduction (Rahman and Singh, 2014; Mahmood et al., 2015; Huang et al., 2016), because high concentrations of the mediator compounds could arouse inhibitory effects on microbial growth and its metabolic activity (Wolf et al., 2009; He et al., 2020).

**FIGURE 3:**
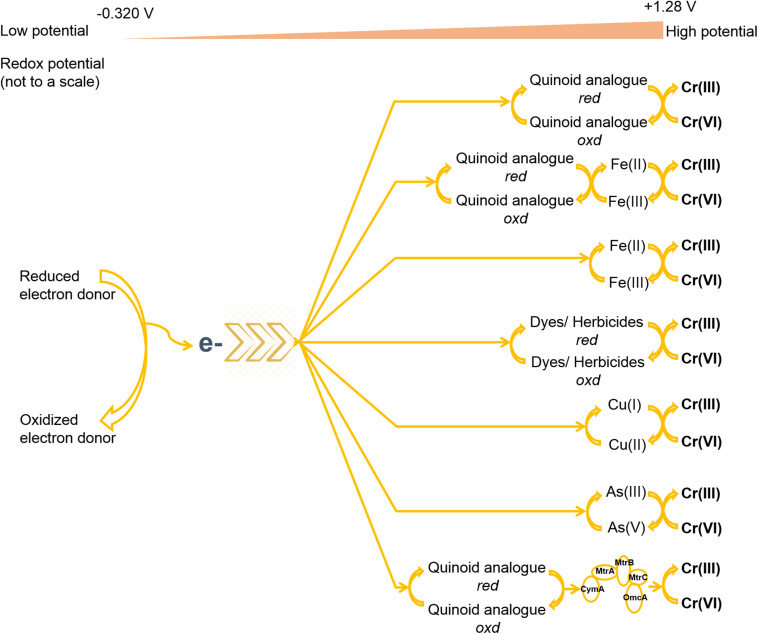
The utilization of various electron donors for Cr(VI) reduction. EVO and ETS mean emulsified vegetable oil and electron transport system, respectively. Sign represents assimilation.

Electron mediators for Cr(VI) reduction belong to a diverse range of organic compounds of foreign origins and endogenous metabolism and can involve some heavy metals (Figure 3; Smutok et al., 2011; Xafenias et al., 2013; Huang et al., 2019; Cao et al., 2020). Often organic mediators are heterocyclic aromatic rings possessing conjugated bonds that enable reduction and rearrangement at biologically accessible reduction potentials (Chai et al., 2019). Various humic substances and their quinoid analogs such as lawsone, menadione, anthraquinone (AQ), anthraquinone-1-sulfonate (α-AQS), anthraquinone 2-sulfonate (AQS), anthraquinone-1,5-disulfonate (1,5-AQDS), anthraquinone-2,6-disulfonate (AQDS), anthraquinone-2,7-disulfonate (2,7-AQDS), 1-chloroanthraquinone (1-CAQ), 1,5-dichloroanthraquinone (1,5-DCAQ), 1,4,5,8-tetrachloroanthraquinone (1,4,5,8-TCAQ), etc., have been popularly used to increase the electron transfer rate for microbial Cr(VI) reduction (Guo et al., 2012; Hong et al., 2012; Huang et al., 2019). Mainly, the carboxyl and phenolic hydroxyl groups present in quinoid compounds of mediators take accountability for the redox conversions between electron donors and the high valence state of the metal (Xu et al., 2020). The position and the presence of chloride ion(s) and other substituents in the aforementioned quinoid compounds also influence electron transfer limitations in Cr(VI) reduction (Hong et al., 2012; Zhang et al., 2014; Dai et al., 2016; Lian et al., 2016). In some investigations, the synergistic associations of Fe(III) minerals and AQDS were involved to deliver an alternative and attractive strategy of enhanced Cr(VI) reduction for co-contaminated sites using microorganisms like *Cellulomonas* sp., *Shewanella oneidensis*, and *Geobacter sulfurreducens* (Field et al., 2013; Meng et al., 2018; He et al., 2019; Liu et al., 2019b). This association employed Fe(III) reduction, and the biogenic product of this reaction, Fe(II), made possible catalysis of Cr(VI) reduction, where AQDS served as an electron carrier between the electron donor and Fe(III). However, the unaccompanied employment of Fe(III) produced lower effect on Cr(VI) reduction because of insoluble nature of the metal ion (Sharma, 2002). In another study, the biomass of henna plant showed a dual role as electron donor and redox mediator for Cr(VI) reduction. A compound, lawsone, present in the biomass displayed rate-enhancing effect on Cr(VI) reduction, whereas the VFAs and H_2_ produced from hydrolysis and fermentation of carbohydrate and protein supplied electrons for reduction (Liu et al., 2010; Huang et al., 2016). Various other organic compounds such as riboflavin, uric acid, dissolved organic matter, etc., have also enhanced the performance of Cr(VI) reduction by shuttling electrons from different microbial metabolisms (Xafenias et al., 2013; Mahmood et al., 2015; He et al., 2020). Another cellular metabolite, pyrrole-2-carboxylic acid (C_5_H_5_NO_2_), also accelerated the bioreduction process in presence of high Cr(VI) concentration by *P*. *phragmitetus* BB (Chai et al., 2019). Interestingly, the EPSs in a sulfur-reducing bacterium, *Enterococcus avium* strain BY7, were also reported to support as an electron carrier for Cr(VI) reduction, where different EPS components such as polysaccharides, proteins, and humic substances exhibited dissimilar electron transfer rates (Yan et al., 2020). Further, the self-assembly of strain BY7 on reduced graphene oxide (rGO) showed enhanced EPS production on bacterial surface for increased removal of Cr(VI). However, the crucial role of rGO in this association involved in reduction of Cr(III) into elemental Cr (a rare species) by lowering electronic potential or activation energy. Several dyes such as methylene blue, neutral dye, dichlorophenolindophenol (DCPIP), meldola blue, and Nile blue have also possessed electron transfer efficiency for Cr(VI) reduction in different fungal and bacterial cells (Smutok et al., 2011; Bai et al., 2018). Simultaneous removal of dyes along with Cr(VI) sets another reputation for their involvement in this reaction. However, such prominence is more suitable, when dyes act as electron donors. Two herbicides, ethyl and methyl viologens, which have been commonly used as mediators for biotransformation of different chemical pollutants, were also employed in Cr(VI) reduction. But the very low redox potential of viologen compounds weakened their roles for Cr(VI) reduction and procured only limited accelerations (Kavita and Keharia, 2012). Some heavy metals such as Cu, Fe(III), and As(III) acting as mediators have also accelerated the enzyme activity for Cr(VI) reduction (details in the following subsection). The role of Cu as a mediator in Cr(VI) transformation is very prominent in various microorganisms. Several Cr(VI) reducers such as *Enterobacter* sp. DU17, *Bacillus* spp., *Pseudomonas putida* KI, *Ochrobactrum* sp. strain CSCr-3, *Brevibacterium* sp. K1, and *Stenotrophomonas* sp. D6, etc., have shown positive effects of Cu on reduction process using different approaches (Camargo et al., 2003; Desai et al., 2008; He et al., 2009; Rahman and Singh, 2014; Ge et al., 2015; Mahmood et al., 2015). Wang et al. (2017b) showed that Fe(III) as an electron shuttle stimulated 1.6-fold more reduction of Cr(VI) in an MFC.

Different mediator compounds have shown different electron transfer competence for Cr(VI) reduction (Wang and Jia, 2007; Liu et al., 2010; Cheng et al., 2019). For example, the addition of AQS greatly improved electron transfer efficiency in *Escherichia coli* BL21, complying 98.5% Cr(VI) reduction, whereas the presence of α-AQS, 1,5-AQDS, AQDS, and 2,7-AQDS could enable only 21–34% metal reduction (Guo et al., 2012). Another investigation reported increased rate of Cr(VI) reduction by *E. coli* K12 using different shuttles following an order of lawsone > menadione > AQS > AQDS (Liu et al., 2010). Even many microorganisms exhibited the ability to utilize multiple electron mediators for the acceleration of Cr(VI) reduction rate (Field et al., 2013; Huang et al., 2019; He et al., 2020). In different studies, redox mediators were also reported to alter the electron transfer pathway of some microorganisms for Cr(VI) reduction (Yan et al., 2014; Dai et al., 2016). The suitable example is the supply of 2-amino-3-chloro-1,4-naphthoquinone-GO (NQ-GO) in *Acinetobacter* sp. HK-1, which shifted and promoted electron transfer from cytoplasmic fraction to membrane counterpart for extracellular Cr(VI) reduction (Zhang et al., 2014). The electron transfer competence of different redox mediators also varies in different microorganisms, where several other factors may administer different stimulation. In many anaerobic conditions, electrons can be shuttled by series of membrane-associated proteins, where the adsorbed Cr(VI) onto cell surface can be subsequently reduced using various membrane-bound reductases (Komori et al., 1989; Wang et al., 1990; Zhu et al., 2008; Belchik et al., 2011; Cao et al., 2020). In *S. oneidensis* MR-1, the quinone/quinol pool in the cytoplasmic membrane transferred the electron from inner membrane across outer membrane through periplasm using network formed by cytochrome c (cyt c), where the outer membrane decaheme cyt c molecules, MtrC and OmcA, played major roles in the transfer of electrons to Cr(VI) on the cell surface using electron shuttles (Belchik et al., 2011).

Immobilization of redox mediators onto different substances for Cr(VI) reduction has also received attention for their better stability, reusability, and persistence. Moreover, lessening the toxic effects of mediator compounds on microbes by their immobilization is also evident (He et al., 2020). Lian et al. (2016) observed nearly 4.5-fold increase in Cr(VI) reduction rate using shuttle facility from 1-CAQ cellulose acetate beads involving bacterium, *Mangrovibacter plantisponsor*. Additionally, many previous MFCs have developed synthetic conductive polymers such as polypyrrole/9, 10-anthraquinone-2-sulfonic acid sodium salt, Si-carbide derived carbon cathode, rutile-modified polished graphite rod, etc., as insoluble redox mediators for improved concurrent Cr(VI) removal and bioelectricity production (Li et al., 2009; Pang et al., 2013; Gupta et al., 2017; Wang et al., 2017b; Li and Zhou, 2019). Table 3 provides information on the measured capacity of different redox mediators for Cr(VI) reduction by different microorganisms.

**TABLE 3:** Acceleration of Cr(VI) reduction by various electron mediators (electron shuttles) using microorganisms.

Microorganisms	Electron mediators	Cr(VI) concentration	Cr(VI) removal				References
			In absence of other additives		In presence of other additives		
			% reduction	Time duration (h)	% reduction	Time duration (h)	
*Bacillus firmus* TE7	As(III)	100 mg/L	100	60	100	48	Bachate et al., 2013
*Bacillus* sp. 3C3	Anthraquinone 2-sulfonate (AQS)	0.5 mM	13.7	72	39.5	72	Hong et al., 2012
*Escherichia coli* BL21	AQS	0.8 mM	21	7.5	98.5	7.5	Guo et al., 2012
*Pseudomonas putida* KI	Hydroquinone	1 mM	68	12	97	12	Mahmood et al., 2015
	Uric acid	1 mM	∼80	18	92	18	
*Enterobacter* sp. DU17	Cu(II)	50 mg/l	∼61	24	91	24	Rahman and Singh, 2014
	As(III)	100 mg/l	∼61	24	75	24	
*Escherichia coli* K12	Lawsone	0.2 mM	6.8	4	97.5	4	Liu et al., 2010
	Menadione	0.2 mM	6.8	4	91.9	4	
	AQS	0.2 mM	6.8	4	79.0	4	
*Acinetobacter sp.* HK-1	2-amino-3-chloro-1,4-naphthoquinone-GO (NQ-GO)	5.0 mg/L	9.5	11	100	11	Zhang et al., 2014
*Pannonibacter phragmitetus* BB	Pyrrole-2-carboxylic acid (C_5_H_5_NO_2_)	3.0 mM	∼8.0	6	∼20	6	Chai et al., 2019

## Other Chemical Influences

**FIGURE 4:**
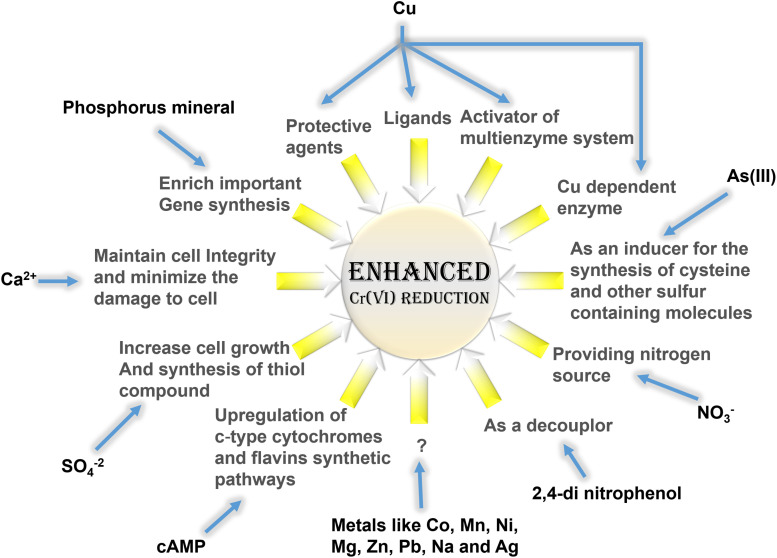
Different pathways for accelerated Cr(VI) reduction using different electron mediators.

The role of chemicals beyond electron donors and mediators for improved Cr(VI) reduction also exists. A variety of chemical affluences acquires diverse mechanisms to advance Cr(VI) reduction process. Even some electron donors and mediators have enhanced Cr(VI) reduction involving other procedures. For examples, heavy metals such as Cu(II), As(III), and Fe(III) that acted as shuttles to accelerate Cr(VI) reduction activity have also upgraded reduction performance by some other effects. Few bacteria recognized Cu as a protective agent for oxygen-sensitive chromate reductase in Cr(VI) reduction (Ibrahim et al., 2012). This metal was also reported to trigger a Cr(VI) reducing enzyme, NAD(P)H-favin oxidoreductase (NfoR), by acting as ligand and changing the enzyme conformation (Han et al., 2017). In a different study, employment of CuO nanoparticles on a biological system activated multiple redox enzymes, and sulfur- and nitrogen-containing proteins for the positive effects on Cr(VI) bioreduction (Yan et al., 2019). A Cu-dependent Cr(VI) reductase is also evident in a bacterium, *Amphibacillus* sp. KSUCr3 (Ibrahim et al., 2012). However, Cu does not necessarily produce encouraging results in all Cr(VI) reducers. Many microorganisms have also experienced the inhibitory effects on Cr(VI) reduction for the effect of Cu (Wang et al., 1990; Park et al., 2000; Pal et al., 2005). A membrane-bound chromate reductase of *Enterobacter cloacae* displayed negative effects after exposure to Cu (Wang et al., 1990). Also, the addition of Cu altered electron transfer pathway and Cr(VI) reduction ability by inhibiting Ni-Fe hydrogenase activity in *Caldicellulosiruptor saccharolyticus* (Bai et al., 2018). Likewise, As(III) also showed varied roles for enhancing Cr(VI) reduction in different microorganisms. A study identified the surplus concentration of As(III) increased the Cr(VI) reduction rate involving *Bacillus firmus* TE7, and complete reduction was observed within 48 h, which was otherwise 60 h (Bachate et al., 2013). This instance sets an example of As(III) as an electron shuttle. In another study, *Enterobacter* sp. Z1 could positively influence the efficiency of Cr(VI) reduction from 64.5 to 92.8% after addition of As(III) within 7 days of incubation (Shi et al., 2020b). In this process, two different mechanisms were proposed for the role of As(III) in enhanced Cr(VI) reduction. In one aspect, As(III) could act as an inducer for the synthesis of cysteine and other sulfur-containing molecules to improve Cr(VI) reduction. Another scheme advised that As(III) as an electron donor might enable redox conversion of Cr(VI). Many other divalent metal ions such as Co(II), Mn(II), Ni(II), Mg(II), Zn(II), and Pb(II) and a monovalent metal ion Ag(I), which usually inhibit chromate reductase activity or produce no significant effect on reduction process, have also promoted Cr(VI) reduction by different bacterial species in various studies (Camargo et al., 2003; Elangovan et al., 2006; Sultan and Hasnain, 2007; He et al., 2009; Liu et al., 2010; Dey and Paul, 2015; Ge et al., 2015; Han et al., 2017; Bansal et al., 2019). However, the exact mechanisms of Cr(VI) bioreduction under the influence of many such metals are yet to be identified. In recent years, the co-presence of another heavy metal, vanadium V(V), has received popularity for their simultaneous removal (Zhang et al., 2012; Wang et al., 2017a, 2018; Shi et al., 2020a). Both Cr(VI) and V(V) have high reducing potential and can be reduced utilizing the same electron sources. But Cr(VI) reduction is preferential and can be enhanced by suppression of V(VI) reduction (Wang et al., 2018; Shi et al., 2020a). Also, this simultaneous removal process involved inhabited approach for V(V) reduction (Wang et al., 2017a). Addition of Ca(II) in the medium weakened the damage of Cr(VI) and promoted Cr(VI) reduction by *Penicillium oxalicum* SL2. The main role of this divalent cation was in stimulating the synthesis of calcium oxalate crystals for maintaining the integrity of cell wall (Luo et al., 2020). Amendment of Na(I) contributed to an increase in the activity of Cr(VI) reduction by the yeast, *Pichia jadinii* M9, where the role of monovalent cation in the bioprocess remained unidentified (Martorell et al., 2012). Even a metabolic inhibitor, 2,4-di nitrophenol (DNP), has also promoted Cr(VI) reduction activity in some Cr(VI) bioreducers (Wani et al., 2007; Alam and Ahmad, 2012; Dey and Paul, 2015). The stimulation of Cr(VI) reduction by DNP is attributed to the fact that its involvement as a decoupling agent could enhance the electron flow in electron transport system, thereby using Cr(VI) as a terminal electron acceptor for maximum reduction under oxygen-limiting conditions (Wani et al., 2007; Alam and Ahmad, 2012). The anionic surfactant, sodium dodecyl sulfate has also influenced the removal efficiency by partitioning of protons due to electrostatic attraction (Nandi et al., 2017). Another anion, SO_4_^–2^ also ensured increased Cr(VI) reduction by improving cell growth in some species (Bonilla et al., 2016; He et al., 2019). In a different way, the synthesis of thiol compounds from SO_4_^–2^ alleviated the oxidative stress of Cr(VI), which accelerated Cr(VI) reduction (Luo et al., 2020). NO_3_^–^-N as the sole nitrogen source also promoted Cr(VI) reduction by *Pseudomonas brassicacearum* LZ-4 (Yu et al., 2016). In another study, the supplement of phosphorus mineral enriched genes related to metal reduction; denitrification; carbon, nitrogen, and phosphorus cycles for the absorption of nutrient synthesis; and electron shuttles for improved Cr(VI) reduction (Ma et al., 2020). Adenylate cyclase expressing *S. oneidensis* MR−1 exhibited enhanced bidirectional EET and Cr(VI) reduction capacities. This was attributed to increased production of cyclic adenosine 3′,5′−monophosphate (cAMP) and cAMP-receptor protein system, which enhanced the gene expression of c−type cytochromes and flavins synthetic pathways for Cr(VI) reduction (Cheng et al., 2020). Similarly, the outer membranes of *Geobacter* spp. with sulfate transporter proteins and cytochromes were critical to metal reduction (Shi et al., 2009; Santos et al., 2015). The enhanced Cr(VI) reduction by microorganisms for the various roles of different chemical additives beyond electron donors and mediators is illustrated in Figure 4.

## Role of Various Chemical Additives in *ex situ* and *in situ* Applications

Development of bioreduction technology for large-scale applications involved a multitude of interactions and biomolecular engineering to multiscale integration. Several key factors are critical to the successful implementation of chemical additives in bioreduction of Cr(VI). Often local physicochemical characteristics present different challenges for the effectiveness of the reduction reaction. For example, reduction of Cr(VI) in contaminated environment can be influenced by the presence of different electron acceptors including O_2_, Fe, Mn, nitrate, etc., which can variably shift the movement of electrons (Sharma, 2002).

Lu et al. (2020) found that natural material, mackinawite, could be effectively operated as electron donors by a neutrophilic chemoautotroph, *Acidovorax* in a batch bioreactor, and the intermediate microbial physiological constituents, VFAs, released from mineral bio-oxidation, could enable the shuttle of electrons to Cr(VI) involving a reducer like *Geobacter*. In an up-flow anaerobic sludge bed reactor, the population of genus *Trichococcus* greatly increased toward the operation period of Cr(VI) reduction using anaerobic sludge, which mainly attributed to fermentation of organic complexes to be available as electron donors, where genera like *Desulfovibrio*, *Ochrobactrum*, and *Anaerovorax* were the main Cr(VI) reducers (Qian et al., 2017). Anaerobic digestion using various electron donors have shown high efficiency of Cr(VI) reduction in different bioreactors (Wang et al., 2018; Zheng et al., 2019). A large laboratory-scale experiment of a 512-day period identified that injection of EVO in Cr(VI)-contaminated aquifer created an acidic and reducing environment, which facilitated Cr(VI) bioreduction by bacteria. Amendment of EVO also greatly influenced microbial community structure and diversity toward the abundance of EVO biodegradation capabilities, which aided supply of reducing equivalents for Cr(VI) reduction (Dong et al., 2018). Additionally, the utilization of bioelectrochemical systems in the form of microbial electrolysis cell (MEC) and MFC is also an innovative route to sustainable bioremediation of Cr(VI)-polluted groundwater (Li et al., 2019b; He et al., 2021). The biogas hydrogen (H_2_) and CH_4_ produced in MEC can serve as electron donors for Cr(VI) bioreduction involving synergism of autohydrogenotrophic genus, CH_4_-metabolizing microorganisms and Cr(VI) reducers (He et al., 2020). Elevated Cr(VI) reduction is greatly convenient in MFC, and simultaneous power generation and removal of co-contaminants were achieved using different variants (Pophali et al., 2020; Zhao et al., 2020a). Yang et al. (2020) showed that compost-derived humic acid along with hematite could promote Cr(VI) reduction by strain MR-1, where the quinonoid and acid groups in organic substances exhibited role as electron shuttle and electron donor, respectively. In a microcosm assay, biostimulation of acetate was effective for anaerobic Cr(VI) treatment in highly alkaline and saline soil of long-term contaminated landfill of León (Guanajuato), Mexico, where a haloalkaliphilic isolate, *Halomonas*, was expected to lead the catalysis of Cr(VI) reduction (Lara et al., 2017). Treatment of Cr(VI)-contaminated soil from tannery site with *Pseudomonas* sp. (RPT) revealed enhancement of Cr(VI) reduction after biostimulation of neem (*Azadirachta indica*) oil cake (NOC), and the application of NOC further improved soil enzyme properties (Govarthanan et al., 2019). Habitually, indigenous microbial communities of different sites greatly influence the reduction process (Lara et al., 2017).

*In situ* amendment of AQSD shifted dynamic state of non-equilibrium transformation of Cr toward accelerated Cr(VI) reduction and inhibited Cr(III) oxidation in contaminated soil (Brose and James, 2010). In a field-scale investigation at Hanford’s 100 area of the United States Department of Energy facility, researchers employed a stable carbon isotope of sodium lactate (13C-labeled) to monitor biostimulation and electron donor fate for Cr(VI) reduction into the high-permeability aquifer comprising gravel and coarse sand sediments (Bill et al., 2019). Several lines of evidence in this study suggested that original ^13^C-lactate underwent some microbial metabolic pathways through total organic carbon (TOC), acetate, and propionate to complete mineralization by serving as electron donors for Cr(VI) reduction. In another study, amendment of organic matter followed by bioaugmentation with a consortium of actinobacteria greatly influenced the Cr(VI) reduction in soil (Lacalle et al., 2020).

## Conclusion and Future Perspectives

The perplexing task of removing Cr(VI) contamination from the environment has led to the development of various bioremediation approaches, particularly the proficient reduction strategies by microorganisms. However, the high efficiency of large-scale microbial Cr(VI) reduction can be limited by the availability of various chemical factors, mainly electron donors, mediators, and cofactors. The oxidation of many suitable chemicals (as electron donors) enables supply of electrons to Cr(VI) to facilitate enhanced reduction. The use of composite matters or combinations of chemical compounds as electron donors grants additional benefits for large- scale application, considering economic aspects. Presence of mediator molecules takes part in improvement of the dynamics of bioreaction. Moreover, several chemicals achieve merits for Cr(VI) bioreduction involving diverse roles other than electron donors and electron mediators. Very often the information on aforementioned chemicals supports their employment for *in situ* and *ex situ* Cr(VI) bioremediation.

Chemical-assisted microbially mediated Cr(VI) reduction is sustainable only when the chemical dosages are low. Otherwise, their high concentrations may cause secondary pollution, impede the ecofriendly approach, and lose prospect of preserving the natural resources particularly for *in situ* applications. Although aforementioned facilities for Cr(VI) bioreduction also exist in environment through natural attenuation at limited efficiencies, in-depth understanding of the governing procedures can device commercial exploitation of the processes for enhanced removal approaches. Therefore, there is a necessity for developing eco-biotechnological schemes that could use unexploited potential of the natural ecosystems for remediation of Cr(VI) contaminated environment. Several key points important to successful application of chemical additives for improved Cr(VI) bioreduction are as follows:

• Identification of novel microbial isolates with better reduction capacity from different environmental samples and their enhanced reducing activity under various chemical affluences are foreseen.
• New knowledge of diverse and specific reduction mechanisms involving chemical consolidation can be constructive in devising effective Cr(VI) treatment.
• The choices of appropriate chemical sources as electron donors, mediators, and cofactors for different microorganisms are important to be recognized.
• Identification of green chemicals for the stimulation of Cr(VI) bioreduction is highly desirable considering the sustainability of ecofunctioning.
• Employment of indigenous chemicals as the substitute for foreign substances may provide better compatibility for the treatment of the contaminated system.
• Employment of wastes generated from other industries may contribute as the cheap alternatives for the Cr(VI) reduction process.
• The electron-donating abilities of different electron donors from composite matters are subject too difficult to analyze, but such integration to biological Cr(VI) reduction for large-scale application and economical virtue states matter.
• The capacity of utilizing endogenous microbial metabolites for reduction process is also rational.
• Chemical integration of donor and mediator compounds often implicates a complex system for Cr(VI) reduction, involving various pathways and processes and multilevel interactions. Such phenomena are also needed to be addressed in-depth.
• The involvement and interaction of numerous compounds by microbial synergy in different intricate pathways have received little attention and therefore warrant further investigations.
• The regular monitoring of microbial growth and Cr(VI) reduction for the treatment of specific chemical utilized for the process is crucial. Moreover, the parameter of soil enzyme activities of the native microbial flora should also be analyzed to identify the various effects of chemical additives.
• The role of many chemicals on Cr(VI) bioreduction is poorly understood, and their characterization relies mainly on past studies. For the same, advanced “omics” technology can add more sight on their specific involvements.
• Variously, genetic manipulation of certain genes of different bioreducers can also empower the microbial ability to exploit various chemical affluences for enhanced Cr(VI) removal.

## Author Contributions

Both authors of this manuscript have made equal contributions to writing, formation of tables and diagrams, editing, and approved it for publication.

## Conflict of Interest

The authors declare that the research was conducted in the absence of any commercial or financial relationships that could be construed as a potential conflict of interest.
